# The Case for Measuring and Reporting Bilingualism in Developmental Research

**DOI:** 10.1525/collabra.233

**Published:** 2019-08-22

**Authors:** Krista Byers-Heinlein, Alena G. Esposito, Adam Winsler, Viorica Marian, Dina C. Castro, Gigi Luk

**Affiliations:** * Department of Psychology, Concordia University, CA; † Department of Psychology, Clark University, US; ‡ Department of Psychology, George Mason University, US; § Department of Communication Sciences and Disorders, Northwestern University, US; ‖ College of Education, University of North Texas, US; ¶ Department of Education and Counselling Psychology, McGill University, CA

**Keywords:** bilingualism, infants, children, hidden moderators, measurement

## Abstract

Many children around the world grow up bilingual, learning and using two or more languages in everyday life. Currently, however, children’s language backgrounds are not always reported in developmental studies. There is mounting evidence that bilingualism interacts with a wide array of processes including language, cognitive, perceptual, brain, and social development, as well as educational outcomes. As such, bilingualism may be a hidden moderator that obscures developmental patterns, and limits the replicability of developmental research and the efficacy of psychological and educational interventions. Here, we argue that bilingualism and language experience in general should be routinely documented in all studies of infant and child development regardless of the research questions pursued, and provide suggestions for measuring and reporting children’s language exposure, proficiency, and use.

Many children around the world grow up learning and using two or more languages. Reported rates of population bilingualism in places such as Europe (67%), Canada (55%), India (25%), and the United States (20%) indicate that bilingualism is both common and growing (Luk, 2017; Office of the Registrar General & Census Commissioner, India, 2001). The current rates for bilingual children are often even higher. For example, in the U.S., 26% of 5–17 year-olds nationwide, and 44% in California are bilingual (Kids Count Data Center, 2018), in Texas, 49% of 0–8 year-olds are bilingual (Park, O’Toole & Katsiaficas, 2017). Bilingualism is particularly prevalent in places where cross-language contact occurs, such as areas with multiple official languages, indigenous languages, or where immigrants settle, pointing to the geographic heterogeneity of bilingualism. We use the term “bilingual” throughout this paper to refer to those learning or using two or more languages, including those who could also be called “multilingual” (Grosjean, 2008, 2015).

Over the past 15 years, evidence has mounted that bilingualism affects not only language development, but a range of other developmental processes, including perception, cognition, brain development, social development, and educational outcomes (Bialystok, 2017; Conboy & Kuhl, 2011; Halle et al., 2014). Yet, information on child bilingualism in developmental studies is not routinely measured and reported. The presence of hidden moderators, defined as unmeasured differences between two purportedly similar studies, can contribute to divergent findings (Stroebe & Strack, 2014; Van Bavel, Mende-Siedlecki, Brady, & Reinero, 2016). For example, in ManyBabies, 67 laboratories worldwide each replicated the same study investigating monolingual infants’ preference for infant-directed speech (ManyBabies Consortium, under revision). The magnitude of infants’ preference was larger for infants whose native language matched the stimuli (North American English) than for those whose native language did not match the stimuli. This illustrates how, if unmeasured, language background can act as a hidden moderator. While this example focuses on difference amongst monolinguals, we argue that bilingualism could have similar effects (ManyBabies data from bilingual infants are forthcoming; Byers-Heinlein et al., 2019). We urge the field of developmental psychology to consider bilingualism as a potentially important hidden moderator, which could impact reproducibility (Open Science Collaboration, 2015). In this paper, we first review evidence suggesting that bilingualism has pervasive, yet still poorly understood, effects on child development. We then give an overview of best practices for defining, measuring, and reporting on bilingualism for studies involving infants and children, even when research questions do not focus on bilingualism or bilingual development.

## Effects of bilingualism on development

Bilingualism affects development across many domains. In this section, we review research showing that bilingualism moderates developmental effects, focusing on research with children (for more extensive reviews, see Bialystok, 2017; Kroll, Dussias, Bogulski, & Kroff, 2011). At the same time, extant research still underestimates the scope of such effects, as there are many areas of development where the impact of bilingualism has yet to be studied.

### Language development

Language outcomes are perhaps the most obvious way that bilingualism affects development. Bilingual children grow to know and use multiple languages, and their development is not akin to “two monolinguals in one person” (Grosjean, 1989). One reason is that children’s experience is divided between their different languages. Moreover, bilingual children must engage in a constant “mental juggling” of their two languages (Kroll et al., 2011), which can present challenges and opportunities for cognition, language representation, and processing (Marian & Shook, 2012). Although a full account of the effects of bilingualism on language development is beyond the scope of this paper, bilingualism has been shown to affect the development of every language system, from speech perception, to phonological development, morphology, vocabulary, and syntax (for reviews, see De Houwer, 1995; Hammer et al., 2014). Thus, bilingualism is essential to document and report in any study that includes language as a predictor, mediator, moderator, or outcome variable.

### Cognitive development

Bilingualism is associated with a range of early cognitive outcomes. Both bilingual infants (Kovács & Mehler, 2009a; 2009b) and children (Barac, Bialystok, Castro, & Sanchez, 2014; Esposito, Baker-Ward, & Mueller, 2013) show advantages in cognitive control, which are modulated by the age of second language acquisition (Barac et al., 2014; Luk, De Sa, & Bialystok, 2011). Monolingual – bilingual differences in childhood have also been reported in reasoning (Byers-Heinlein & Garcia, 2014), stimulus encoding (Singh et al., 2015), and memory (Brito & Barr, 2012, 2013). The fact that the existence and/or size of bilingual cognitive advantages are disputed (Duñabeitia et al., 2014; Paap, Johnson, & Sawi, 2015) only enhances the need for systematic measuring and reporting of child bilingualism, so researchers can understand the mechanisms underlying observed effects.

### Perception

Work on perception in bilingual children has largely focused on speech, with many studies reporting monolingual – bilingual differences as early as infancy (Byers-Heinlein & Fennell, 2014). Bilingual adolescents and adults also show different patterns of speech perception and encoding, even for low-level information such as the fundamental frequency of speech syllables (Krizman, Marian, Shook, Skoe, & Kraus, 2012). Integration of auditory and visual information is affected by bilingualism: bilinguals are less susceptible to illusions that fuse asynchronous non-linguistic auditory and visual stimuli into a single percept (Bidelman & Heath, 2018), but are more susceptible to such illusions with mismatching audiovisual speech syllables (Marian, Hayakawa, Lam, & Schroeder, 2018). There are also effects of linguistic and cultural immersion for how adults perceive and process color, even in pre-attentive tasks (Athanasopoulos, Dering, Wiggett, Kuipers, & Thierry, 2010), as well as for how speakers of different languages process the visual world (Chabal & Marian, 2015) and perform in visual search tasks (Chabal, Schroder, & Marian, 2015). In younger bilinguals, research on perception beyond speech and language has been limited, but domain-general effects of bilingualism on early perception seem likely given the emerging evidence from older groups.

### Brain development

Bilingualism also sculpts the brain’s functional and structural organization (Hayakawa & Marian, 2019). For example, bilingual infants show different brain responses to native and non-native speech sounds than monolingual infants (Conboy & Kuhl, 2011; Garcia-Sierra et al., 2011), and bilingual children recruit different brain areas during sentence processing (Jasinska & Petitto, 2013). Adult work shows that the age of acquisition of a second language affects the brain’s language networks (Berken, Gracco, & Klein, 2017), and functional connectivity (Kousaie, Chai, Sander, & Klein, 2017). Moreover, bilingualism also affects the structure of both grey (Andrea et al., 2004; Ressel et al., 2012) and white matter (Kuhl et al., 2016) in adults. Patterns of structural differences appear to depend on whether two languages were acquired simultaneously from birth, or sequentially before age five (Berken, Gracco, Chen, & Klein, 2015), suggesting that timing of bilingualism interacts with brain development.

### Social development

Bilingualism affects how children interact with and learn from others. For example, compared to monolinguals, bilinguals are more willing to be friends with other bilinguals (Byers-Heinlein, Behrend, Said, Girgis, & Poulin-Dubois, 2016), put more weight on social cues during learning (Yow & Markman, 2011, 2014), are advanced in their theory of mind (Goetz, 2003; Kovács, 2009), and show more sophisticated understanding of social groups (Dautel & Kinzler, 2018; Liberman, Woodward, Sullivan, & Kinzler, 2016). Moreover, there are bi-directional links between bilingualism and children’s social skills, in that bilingual children may have stronger social skills than monolinguals (Han, 2010), and children who are initially more socially skilled are more likely to become bilingual themselves (Winsler, Kim, & Richard, 2014). Finally, bilingual children are often bicultural (Grosjean, 2015), and thus they must negotiate between two often competing sets of cultural expectations in the contexts in which each language is used (Halle et. al., 2014). As a result, compared to monolinguals, they can have more complex cultural identities (Mills, 2001), and may show different reasoning about nationality (DeJesus, Hwang, Dautel, & Kinzler, 2018).

### Educational outcomes

Bilingualism is also related to enhanced educational outcomes for students, but in complex ways, given that in some areas (e.g., the United States) bilingualism is correlated with factors negatively associated with achievement such as poverty, ethnic minority status, immigrant status, and limited proficiency in the language of schooling (Genesee, Lindholm-Leary, Saunders, & Christian, 2005). Controlling for these confounding variables, balanced bilingual students who are proficient in the school language show better educational outcomes compared to monolinguals (Medvedeva & Portes, 2016). For example, in the U.S. context, once former English language learners reach full proficiency in English (while maintaining their first language), they often academically outperform both monolingual English-speaking children, and students who are not yet proficient in English (Ardasheva, Tretter, & Kinny, 2012; Halle, Hair, Wandner, McNamara, & Chien, 2012). Multiple sociocultural factors also come into play, including access to high quality education, first language support, and social status of the first language (Castro et al., 2013), but clearly it is useful to know the bilingual language status of participants when examining educational outcomes.

## Bilingualism as a hidden moderator

A hidden moderator exists when an unmeasured factor varies between studies that can change the effect of interest. Given the evidence reviewed above, bilingualism changes developmental processes and outcomes. Moreover, emerging research suggests that, in some cases, even fairly minimal exposure to a second language can affect performance on experimental tasks (Fan, Liberman, Keysar, & Kinzler, 2015; Howard, Carrazza, & Woodward, 2014). Yet, bilingualism is not systematically measured or reported in many developmental studies, and is operationalized in different ways when it is (Surrain & Luk, 2017; see Byers-Heinlein et al., 2018 and Esposito et al., 2019 for detailed guidelines for measuring bilingualism). Given that some countries, cities, neighborhoods, and schools have larger numbers of bilinguals than others, labs in different locations are likely to have different proportions and types of bilingual children in their samples. When information about language background is not gathered and reported, we are missing opportunities to understand developmental phenomena and account for divergent results.

As an example, imagine two labs that conduct parallel studies to test the effects of an intervention on an educational outcome, without considering that some of their participants could be bilingual. Lab 1 finds that the intervention improves educational outcomes, while Lab 2 finds that it does not. This would be an inconsistency in the literature – a failure to replicate. But what if Lab 1’s sample contains few bilinguals (it is located in a small, largely monolingual college town), while Lab 2’s sample contains many bilinguals (it is located in a linguistically diverse city)? If the intervention is a cognitive training program, it may be less effective for bilinguals than monolinguals, because bilingualism already enhances certain cognitive capacities. Or if the outcome is English vocabulary size, such a measure might be less valid for bilinguals because their vocabularies are distributed across two languages (for evidence from school-aged children see Bialystok, Luk, Peets, & Yang, 2010, for evidence from toddlers see Core, Hoff, Rumiche, & Señor, 2013). In both cases, bilingualism is a hidden moderator, obscuring the nature of the relationship of interest.

Currently, it is impossible to know how often the hidden moderator of bilingualism is behind inconsistencies and failures to replicate in developmental studies. However, given the evidence reviewed above of the many ways that bilingualism affects development, it is a variable that warrants greater attention. Bilingualism can affect research in multiple ways: directly (e.g., affecting scores on a sentence completion task), indirectly (e.g., the validity of an IQ test administered in a single language), or incidentally (e.g., task instructions given in a particular language). We propose that developmental researchers consistently measure and report bilingualism in their samples, whether or not language or bilingualism are of central interest. In the next section, we review two types of variables that should be reported: child-level variables such as the child’s language history and language proficiency, and context-level variables about the child’s family background and the wider community context (see Table 1).

## Child-level variables

Bilingualism is a multi-dimensional construct related to individuals’ language history, language use, and language proficiency (Luk & Bialystok, 2013). While there is some disagreement as to whether bilingualism is better understood as a categorical variable or a construct that occurs along a continuum (Luk & Bialystok, 2013), a bilingual can be roughly defined as a person who uses two or more languages in everyday life (Grosjean, 2008). Under a categorical approach to bilingualism, there can be some disagreement as to what threshold of exposure or use is necessary for a child to be considered “bilingual.” For example, studies of bilingual infants typically focus on language exposure, and have used definitions that ranged from exposure to each language 10–90% of the time, to exposure to each language 35–65% of the time (Byers-Heinlein, 2015). Indeed, sometimes these definitions overlap with criteria for monolinguals (e.g., infants exposed to a single language more than 80% of the time). In older children, language use and/or proficiency are often used to define bilingualism, either instead of or in addition to language exposure (Paradis, Emmerzael, & Duncan, 2010).

Nonetheless, it is well-established that the number of and which particular languages a child speaks, the age they started learning them, how often they hear and speak them, and their proficiency in these languages all affect developmental outcomes, as well as performance on psychological measures and laboratory tasks. We recommend that developmental researchers whose work does not focus on bilingualism acknowledge these potential sources of variation by routinely measuring and reporting children’s language background in as much detail as feasible. When samples are complex and diverse, online information can supplement in-text summaries. Below, we discuss four key child-level variables: languages of exposure, onset of exposure, amount of exposure and use, and proficiency.

### Languages of exposure

Studies should report the languages of exposure for infants and children, and report use for children. In cases where children are hearing multiple varieties (i.e., accents or dialects) of the same language, this may also be valuable information to include (e.g., Floccia, Luche, Durrant, Butler, & Goslin, 2012). Patterns of exposure to the same language with different accents can also change developmental outcomes (Buckler, Oczak-Arsic, Siddiqui, & Johnson, 2017). Specific terms are preferred over general ones, for example “Mandarin” is preferred to “Chinese”.

### Onset of exposure

Children vary in terms of when they began acquiring different languages. Nearly all children acquire at least one native language from birth. Simultaneous bilingual children acquire two languages from birth, and sequential bilingual children begin acquiring a second language sometime after birth. Historically even in studies focusing on bilingualism, age of acquisition has been reported with relatively little precision, (e.g., terms such as “early bilinguals”). However, the precise timing of acquisition of each language can impact development, for example, the difference between learning a language from birth versus later in school (Choi, Black, & Werker, 2018; Sebastián-Gallés, Echeverría, & Bosch, 2005). Empirical and theoretical work points to the need to be as precise as possible when reporting acquisition onset, and whether it was interrupted at some point (e.g., exposure or use of a language stopped due to relocation).

### Amount of exposure and use

Children vary widely in how much they hear and use each of their languages. Some children are clearly monolingual, with nothing but minimal incidental exposure to additional languages. Others hear and use two or more languages to varying degrees. The average and range of exposure to each language is tightly linked with performance on experimental tasks and language outcomes (Byers-Heinlein, Morin-Lessard, & Lew-Williams, 2017; Hoff et al., 2012; Marchman, Martínez, Hurtado, Grüter, & Fernald, 2016). Similar patterns are found with language use: children who use a language more have better outcomes in that language (Bohman, Bedore, Peña, Mendez-Perez, and Gillam, 2010). While the underlying shape of the function linking exposure and use to outcomes is not yet well understood, studies have found systematic differences between monolinguals, bilinguals, and children who are incidentally exposed to non-native languages (Akhtar, Menjivar, Hoicka, & Sabbagh, 2012; Howard, Carrazza, & Woodward, 2014). Therefore, documenting language experience and use is necessary for children from all language backgrounds, particularly for bilingual children.

### Proficiency

For monolingual children, age is an adequate proxy to determine the expected range of language proficiency. However, bilinguals often have unequal proficiencies in their languages, and there can be a large dissociation between chronological age and language proficiency. Moreover, bilingual children’s language knowledge can be unevenly distributed, for example, knowing some words in one language but not the other (e.g., academic vocabulary in the school language, colloquial vocabulary in the home language; Bialystok et al., 2010). In addition, bilinguals’ proficiency is highly dynamic, and may either increase or decrease over time as patterns of language exposure and use change (Winsler, Díaz, Espinosa, & Rodr–guez, 1999). It is particularly important to measure and report proficiency when this might influence performance on an outcome variable (e.g., a verbal component of an IQ test): researchers should be extra cognizant about children’s proficiency in the language of testing. See Esposito et al. (2019) and Peña and Bedore (2018) for detailed recommendations for measuring proficiency in bilingual children.

## Context-level variables

Community and family context have been long recognized as important for understanding children’s development. Indeed, since November 2014, the journal *Child Development* has required the reporting of “Socioeconomic status, language, family characteristics, specific location information, etc.” in addition to previous requirements to report “participant age, gender, and race/ethnicity” (Society for Research in Child Development, 2014, 2018). Information about language use in the community, family, and educational settings provides important context about bilingual (and other) development (Castro, 2014), and we argue that these variables should also be reported. Moreover, these context-level variables can also provide insight into participants’ cultural backgrounds, which is important as many bilingual individuals are also bicultural (Grosjean, 2015), which in itself could act as a hidden moderator.

### Community context

Community matters to language development because children show better language outcomes in languages they hear widely in their environments, particularly from native speakers (Gathercole, 2014; Place & Hoff, 2010). Yet, less than 30% of studies comparing monolinguals and bilinguals report the larger sociolinguistic context (Surrain & Luk, 2017). Both primary and additional languages spoken in the community are important for understanding the developmental context.

### Family context

Factors such as immigration history, racial/ethnic background, country of birth, and language of schooling of parent and child are particularly relevant in the case of bilinguals. This is because they are related to the ways in which language is used, and in turn, language learning and outcomes. Unlike monolingual families, bilingual families differ in which languages are spoken by whom, when (Castro, 2014), and specifically to the child (Espinosa et al., 2017), and undergo language changes as different individuals join or leave the household (Verdon, McLeod, & Winsler, 2014). The socio-economic status (SES) of the family is also important because, in some communities, bilinguals are heterogeneous in SES, while in others, bilinguals may differ systematically from monolinguals (Morton & Harper, 2009). Studies should measure and report SES (e.g., by using a proxy variable such as maternal education) separately for monolingual and bilingual participants. If SES diverges across populations, it can be included as a covariate, or considered in the interpretation of any observed monolingual – bilingual differences.

### Educational context

The language used in childcare and schools varies considerably across children and communities (Goldenberg, 2015; Kim, Hutchison, & Winsler, 2015). Some children attend school exclusively in the majority language, which may or may not be their first language. Other children attend programs that support both their languages, such as two-way immersion programs. Still other children may learn a minority language not spoken in the family (i.e., a nanny who speaks another language, or an immersion program in an additional language). These different educational contexts will affect children’s exposure to and learning of different languages. Moreover, different proficiencies in the language of schooling give children different opportunities to access academic content. Thus, the language(s) children hear and use in educational settings should be reported.

## How to measure bilingualism

Parents, teachers, and older children themselves will often be the best sources of information about child-level, family, and educational variables. For many research purposes, it may be sufficient to add a few carefully-worded questions to existing questionnaires. We provide examples of these types of questions in Table 1. The level of detail of information to gather and report will depend on the specific goals and methods of the study, as well as the age of the participants (see Byers-Heinlein et al., 2018 and Esposito et al., 2019 for more detailed guidelines). Researchers will need to select and adapt questions to their own research questions, study protocol, and populations, especially when testing in time-limited situations such as schools or museums. Even asking for a postal/zip code and languages spoken at home and school and by whom would be an important step forward.

For studies specifically focused on language, most researchers advocate for the use of detailed structured interviews with individuals familiar with the child, who can provide information about the languages the child hears and speaks in different contexts, when the exposure began, and how often each language is heard and used, as well as other family-level variables (Byers-Heinlein et al., 2018). This approach has high validity, based on comparisons between parent-report measures and daylong home language recordings (Orena, Byers-Heinlein, & Polka, under review). Several instruments and approaches are available, and are ideally administered by culturally-sensitive, bilingual researchers (Cattani et al., 2014; DeAnda, Bosch, Poulin-Dubois, Zesiger, & Friend, 2016; Liu & Kager, 2016; Paradis, Emmerzael, & Duncan, 2010; Peña, Gutierrez-Clellen, Bedore, & Iglesias, 2018; see also Does et al., 2018, for a broader discussion of research staff demographics). Researchers can refer to Esposito et al. (2019) for a more detailed discussion of in-depth measures of bilingualism.

For community context, local and national governments typically provide information online about languages used in the community. For example, the U.S. Census Bureau’s American FactFinder (https://factfinder.census.gov/faces/nav/jsf/pages/index.xhtml) provides language diversity statistics for state, county, city, town, or zip code provided as a simple search. In Canada, GeoSearch maintained by Statistics Canada provides a similar tool for capturing language diversity from the census data (https://www12.statcan.gc.ca/census-recensement/2016/geo/geosearch-georecherche/index-eng.cfm). In Europe, EuroStat provides summary statistics of learning and knowledge of foreign languages, with links to the original data source (e.g., https://ec.europa.eu/eurostat/statistics-explained/index.php?title=Foreign_language_learning_statistics#Primary_education).

## Conclusions

Increasing numbers of infants and children worldwide grow up bilingual. We now understand that bilingualism affects development across a broad range of cognitive, social, and neural processes and outcomes, far beyond the domain of language. Here, we have argued that bilingualism may act as a hidden moderator in studies of child development. Routinely measuring and reporting bilingualism whether or not language and/or bilingualism are the research focus will improve the replicability of research, and our understanding of child development.

## Figures and Tables

**Table 1:** Recommended variables with examples for describing bilingualism in infants and children.

	Variable	Description	Sample questions/how to measure	Example text for *participants* section
**Child**	Languages of exposure	The languages the child hears	What language(s) does your child hear/speak at home? At school?	All 72 children were acquiring French, and 29 had regular exposure to an additional language. Additional languages included Arabic (*n* = 15), Spanish (4), Catalan (3), Portuguese (2), and 1 each of Basque, Cantonese, Dutch, Hungarian, and Yoruba.
	Onset of exposure	Age at which child began hearing each language	At what age did your child begin regularly hearing [languages]?	Twelve children were exposed to both Spanish and Catalan simultaneously from birth. Thirty-six were initially exposed primarily to Spanish and began hearing Catalan upon entering preschool at age 3.
	Amount of exposure and use	How much the child hears each language, currently and/or cumulatively	How many hours per day/week/percentage of the time does your child hear/speak [languages]?	Infants were exposed to each of their two languages between 25% and 75% of the time since birth. Exposure to the most-heard (dominant) language averaged 65% (range: 50–75%) and exposure to the least-heard (non-dominant) language averaged 35% (range: 25–49%).
	Proficiency	Child’s level of ability in comprehending, speaking, reading, and/or writing the language.	In comparison to other children of the same age who are native speakers of [language] rate your child’s ability to understand/speak/read/write [language]. [Likert scale]	Children’s comprehension of Mandarin was rated by parents as high, with children receiving an average score of 8.3 in comprehension (range: 7–10), where 0 was “no ability to comprehend Mandarin” and 10 was “excellent ability to comprehend Mandarin”.
**Context**	Community	Official or predominant societal languages Other languages spoken widely in the community	*Typically available from government websites, census data*.	Children were growing up in Montreal, a city where both French and English are regularly spoken in everyday life. Fifty-nine percent report fluency in both languages.
	Family	Which languages are spoken by whom Family background: immigration, education, ethnicity Socio-economic status	Were the child’s caregivers born in [country of testing]? If not, what year did they arrive? In what language did [caregivers] receive the majority of their education? What ethnic/cultural group(s) does your family identify with? What is the mother’s highest level of education?	Children were growing up in families where Spanish was the primary home language, although in 30% of families there were older siblings who spoke both English and Spanish at home to the child. Families were typically from mid- to lower SES backgrounds: 80% of mothers had a high school education or less, 20% had completed at least some postsecondary education. All parents, and 30% of children, were born outside of mainland United States. Families’ place of origin included Puerto Rico (45%), Mexico (20%), Cuba (20%), Argentina (10%), and Peru (5%).
	Education	Languages spoken and taught in school Approach to language teaching	What is the primary language(s) of school instruction? Are any other languages taught (which)? How many hours/week are they taught? *For in-school testing, this can be obtained from teachers/administrators*.	Children were in their first year of a French immersion program, where French was used for 80% of instructional time, and English was used for 20% of instructional time.
